# The Changing Landscape of Ankle Fractures in the Era of Electric Micromobility: A Systematic Review

**DOI:** 10.7759/cureus.96812

**Published:** 2025-11-13

**Authors:** Suvank Rout, Souvagya Rout, Ronish Patidar

**Affiliations:** 1 College of Medicine, Topiwala National Medical College and Bai Yamunabai Laxman Nair Charitable Hospital, Mumbai, IND; 2 Trauma and Orthopaedics, Colchester General Hospital, Colchester, GBR; 3 Radiology, First Faculty of Medicine, Charles University, Prague, CZE; 4 Orthopaedics, Indira Gandhi Medical College and Hospital, Shimla, IND

**Keywords:** ankle and foot, ankle fracture management, electric scooter accidents, electric scooter injury, e-scooter injury

## Abstract

Electric bicycles (e-bikes) and electric scooters (e-scooters) have been rapidly adopted in urban environments in the United Kingdom and various Western countries. This has introduced a new mechanism of injury in these environments. Whilst the initial research studies focused on head injuries, upper limb injuries and overall epidemiology, the specific patterns regarding lower limb orthopaedic injuries, especially ankle fractures, are not well defined.

This systematic review aims to characterise ankle fracture patterns due to e-bike and e-scooter use and assess whether the incidence and severity have worsened since adopting these devices. We performed a systematic literature review in PubMed for articles published between 2015 and 2025. This timeframe was selected as micromobility adoption was uncommon prior to 2015. Our initial search yielded 83 studies; after screening and eligibility assessment, 11 studies were included for final analysis. From the studies, we were able to identify that there was a distinct injury pattern associated with e-scooters, i.e., isolated ankle rotational fractures from low-energy falls. Data from UK Government sources initially indicated that e-scooter users primarily experienced low-energy injuries, while e-bike users, although reported less frequently, seemed to suffer more high-energy fracture patterns due to higher speeds. However, recent data suggest that the frequency and severity of injuries from e-scooter accidents are increasing, with more cases exhibiting high-energy mechanisms. UK-specific data, though limited, align with global trends, indicating a significant and growing burden on Emergency Departments and Orthopaedic services, with a notable proportion of injuries requiring operative intervention. In comparative studies, e-bike crashes were less frequently linked to ankle fractures. All data indicate a sharp rise in overall micromobility injuries over recent years.

Ankle fractures from electric micro-mobility devices represent a significant and evolving public health issue. While consistent with international findings, the current evidence from the UK is insufficient. There is a pressing need for prospective, multicentre studies and national registry data to define these injury patterns better and inform targeted public safety measures and orthopaedic preparedness.

## Introduction and background

The use of micro-mobility devices such as electric scooters (e-scooters) and electric bikes (e-bikes) has risen drastically since 2018 in the United Kingdom (UK). These micro-mobility devices have been promoted as a green and convenient solution for "last-mile" travel. This is since e-scooter and e-bike rental schemes were introduced in July 2020, amid the pandemic, in approximately 30 cities [1]. Micromobility (e-scooters/e-bikes) use has risen steeply in urban areas since 2018-2020. Multiple trauma centres report increased presentations and operative caseload linked to these devices [2]. Initial epidemiological studies primarily focused on the overall incidence of e-scooter and e-bike injuries and the concerning prevalence of craniofacial and head trauma. Whilst craniofacial and upper limb injuries are well documented, foot and ankle injuries have been a key driver of orthopaedic presentations and admissions to hospitals, often requiring surgical intervention. Studies have shown that ankle fractures are the most common fracture patterns [3]. The ankle, a complex weight-bearing joint, is biomechanically vulnerable to high-impact, torsional forces commonly experienced during micro-mobility incidents [3].

Studies report that e-scooter-related emergency visits rose from ~4,900 in 2014 to ~29,600 in 2019 [4]. There was a 2371% increase in e-bike-associated fracture cases in the United States of America between 2019 and 2023 [5]. UK hospital services and national data (Department for Transport/UK e-scooter factsheets) show increasing numbers of serious lower-leg/ankle injuries associated with e-scooters. Studies have reported that the e-scooter-related casualty visits rose from 384 in 2020 to 1,390 in 2025 [6,7].

A critical gap in the current literature is a dedicated synthesis of the specific patterns of ankle fractures and an analysis of whether these injuries have increased in severity over time. As vehicle speeds increase and rider demographics expand to include less experienced users, there is a compelling clinical suspicion that the nature of ankle trauma is worsening [8]. This systematic review therefore aims to synthesise the existing evidence on ankle fracture patterns resulting from e-scooter and e-bike use, analyse temporal trends in the incidence and severity of these fractures since the widespread adoption of these devices and provide a specific sub-analysis of UK-based data to inform national policy and clinical practice.

## Review

Methods

We conducted a systematic literature search using PubMed and related sources from 2015 to 2025, employing the terms "Electric Scooter", "e-scooter", "Electric Bike", "e-bike", "micromobility", "ankle fracture", "ankle injury" and "lower extremity injury". We followed the Preferred Reporting Items for Systematic Reviews and Meta-Analyses (PRISMA) guidelines for systematic reviews. Two reviewers screened titles/abstracts and full texts, extracting data on study design, population, fracture types, severity, and temporal trends. The methodological quality of the included studies was assessed using the Joanna Briggs Institute (JBI) checklist for systematic reviews and research syntheses [9]. Discrepancies between authors were resolved by discussion.

Inclusion Criteria

We included studies reporting on ankle fractures or lower extremity injuries with ankle fractures resulting from e-bike or e-scooter use. These included observational studies, case series, and randomised controlled trials. Studies with ≥10 patients reporting specific ankle fracture outcomes were also included.

Exclusion Criteria

We excluded single case reports or non-specific injury reports. Case reports were also excluded from this study unless they were part of a larger series.

**Figure 1:**
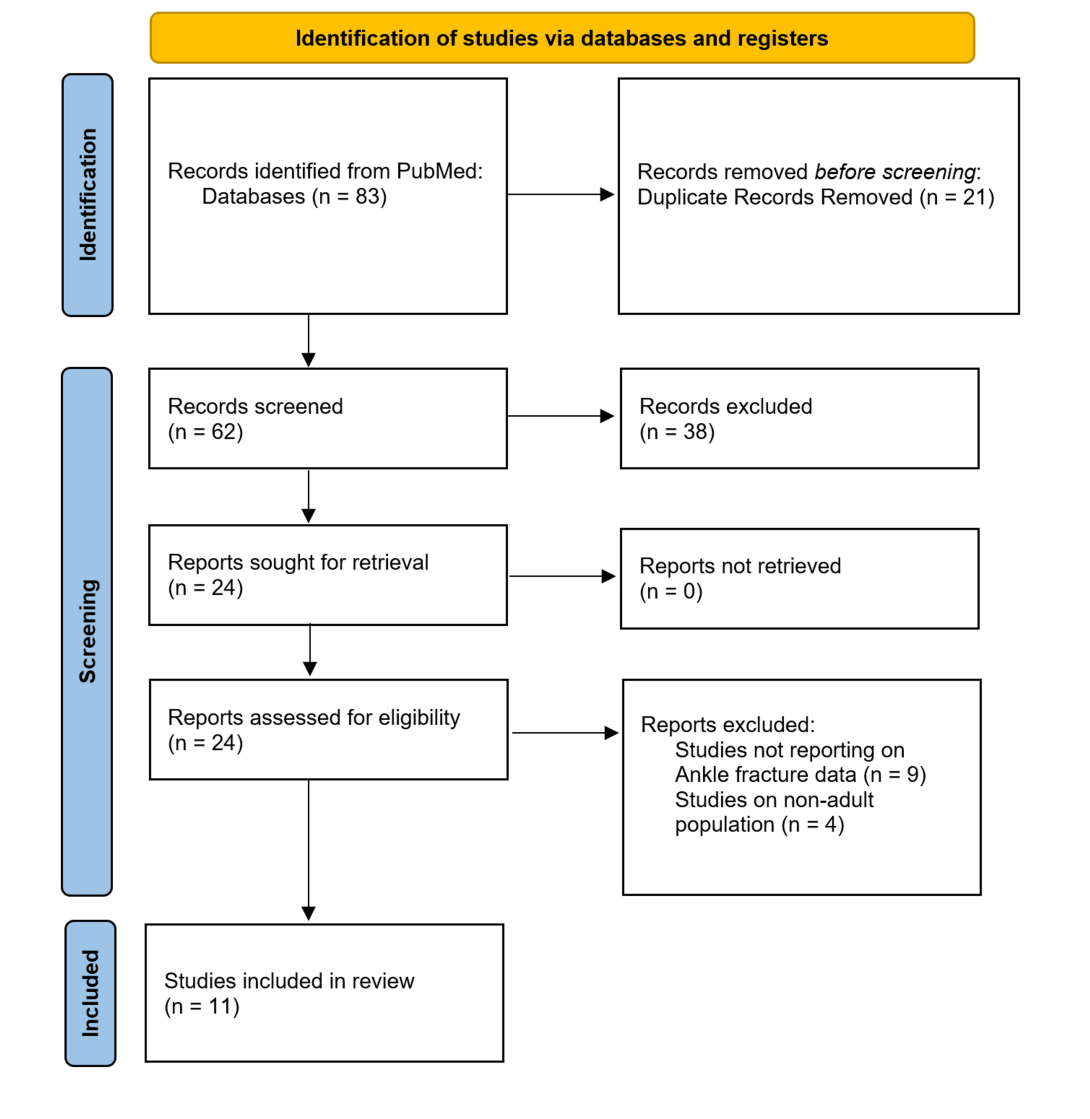
Preferred Reporting Items for Systematic Reviews and Meta-Analyses (PRISMA) flowchart

The initial literature search identified 83 records. After deduplication and application of exclusion criteria, 11 studies were identified. The final pooled set comprised retrospective cohorts from trauma centres and registry analyses, and no randomised trials were found, which have been summarised in the PRISMA flowchart (Figure 1).

Results

In the initial literature search, we found 83 studies using the keywords. After applying the exclusion and inclusion criteria, the authors identified 11 studies.

A level 1 trauma review by Fisher et al. reported 16 ankle fractures among 136 e-scooter/e-bike injuries (11.7%). There was a 33.3% incidence of associated posterior malleolar fractures in the spiral tibial shaft fractures, 31.0% of posterior malleolar involvement and 18.8% of isolated vertical medial malleolar fractures in the ankle fractures, and 61.5% of posterior comminution in the tibial plateau fractures [3]. A Korean tertiary hospital series of 563 e-scooter injuries, by Kim et al., identified that the lower limbs were the most common injury site. Among the fracture group (20 cases), ankle fractures (nine cases) were most common, including pronation external rotation type 4 injuries (four cases) and pilon fractures (two cases). Five patients (25%) had open fractures, and 12 patients (60%) underwent surgical treatment [4]. Cruz et al., in their multicentre study in London, reported on 83 e-scooter patients with 105 injuries. There were 93 fractures, of which 39 (41.9%) were lower limb and 12 were open fractures (12.9%) [10]. The most common injuries noted in the study were upper limb injuries (56%), mainly affecting the radius (24% of all injuries) [10]. Flaherty et al. found in their retrospective analysis of 20 patients with 27 foot and ankle fractures, a high rate of complex injuries. Eight patients suffered fracture dislocations, four sustained open fractures, and 45% (9/20) of patients required surgical treatment [11]. The Swedish Fracture Register reported 1,874 fractures in 1,716 patients from e-scooter accidents (April 2019-December 2022), where only ~5% were ankle fractures. Most of the injuries noted were upper limb injuries (59% of the cohort) [12]. Bentham et al. performed a cross-sectional review of radiology exams of 340 patients (568 studies) related to e-scooter injuries from 2015 to 2022, and noted 126 musculoskeletal injuries. They found that radial head injuries were the most common injury (n=27/21%). Foot and ankle fractures contributed to (n=14) 11% of all musculoskeletal injuries [13]. Metry et al. found that 65% of all patients suffered injuries to their extremities, with 22.1% being open fractures. In their study, 45% (n=31) of all injuries affected the lower limbs [14]. One multicentre cohort study in Switzerland by Rauer et al. compared lower extremity fractures from e-bikes, regular bikes and motorcycles. Rauer et al. found that the fractures of the foot (n=3) and proximal tibia (n=3) were the most commonly injured anatomical structures of the lower limb. No ankle fractures were caused by e-bikes in their study [15]. In their Newsroom press release, the American Academy of Orthopaedic Surgeons (AAOS) stated that micromobility devices such as e-bikes and e-scooters can result in forces causing unusual fractures, which are not seen with standard bikes [16].

Kim et al. noted a year-over-year increase in micromobility injuries in their four-year series [4]. Senel et al. observed that fracture rates increased in the year 2022-2023 when compared to previous years [17]. The United States Consumer Product Safety Commission (CPSC) shows that injuries associated with all micromobility devices increased nearly 21% in 2022 from 2021 [18]. Micromobility-related injuries have trended upward since 2017, increasing an estimated average 23% annually [19]. In their cross-sectional study, Bentham et al. noted an increase in micromobility-associated injuries between 2015, with a marked increase in injuries after an e-scooter sharing scheme began (October 2020), with summer peaks [13]. Shichman et al., in their study in Tel Aviv, found a six-fold increase in e-scooter-related injuries presenting to the ED, from an average of 26.9 injuries per month before the introduction of shared e-scooter services in August 2018 to an average of 152.6 injuries per month after its introduction [19].

Cruz et al. found that e-scooter accidents cause high-energy limb trauma, resulting in 25 patients (30.1%) requiring an operation and 29 (34.9%) requiring hospital admission [10]. Kim et al. noted 12 patients (60%) underwent surgical treatment for their lower limb injuries, which included pronation-external rotation type IV injuries, pilon fractures and open fractures [4]. Flaherty et al. noted that out of all injuries associated with e-scooters, 45% of patients required surgical intervention [11]. Ishmael et al. found in their study that 73 patients required operative treatment in just the first two years of e-scooter use in their community, with operative injuries occurring throughout the skeletal system, and several injuries were associated with high-energy trauma [20]. Vasara et al. noted that 60 (13%) patients required hospital admission after their e-scooter accident, and five patients who had suffered ankle fractures all required surgical intervention [21]. Bascones et al. found 117 different fractures in 105 patients from the 167 patients included in their study. A total of 105 (63%) patients also required hospital admission due to the high-energy mechanism of injuries sustained [22]. Notably, the demographics skew young (mean ~26-34 years) and male, often involved in summertime or weekend crashes [4,17].

Summarising the evidence, e-scooter crashes often injure feet/ankles and upper limbs. The common patterns are pronation-external rotation ankle fractures with posterior malleolar or vertical medial malleolar involvement. In contrast, ankle fractures are rare in e-bike accidents, but when they occur (often as dislocations), they imply high-energy transfer similar to motorcycle injuries. A summary of all the studies is provided in Table 1.

**Table 1:** Comparative Review of Recent Micromobility Injury Studies

Author	Location	Duration of Study	Study Design	Population (Number of Injuries)	Key Finding
Fisher et al. [3]	New York, USA	2021	Retrospective Trauma Registry	119 riders (136 injuries)	The study focused on lower extremity injuries, finding that ankle fractures were the most common (16.7%), followed by tibial shaft fractures, with a high rate of complex joint fractures.
Kim et al. [4]	Korea	2020-2024	Retrospective Cohort	563 patients (52 ankles)	This study highlighted lower-limb injuries, noting that the ankle was the most frequently injured site and that a significant 25% of these were severe open fractures.
Cruz et al. [10]	London, UK	2018-2020	Multicenter Trauma Study	83 patients (105 injuries)	This study captured a near-even split of injuries, with 56% affecting the upper extremity and 41.9% affecting the lower extremity. E-scooter accidents cause high-energy limb trauma, resulting in 30.1% patients requiring a surgical intervention
Flaherty et al. [11]	London, UK	2020	Retrospective Case Series	20 patients (27 injuries)	This case series focused on lower extremity trauma, finding that 86% of patients with foot and ankle fractures required surgery.
Hernefalk et al. [12]	Sweden	2019-2022	Registry Based Cohort	1,716 patients (1,874 fracs)	This large registry cohort was dominated by upper-extremity injuries (40%), with fractures of the distal radius (wrist) being the single most common injury at 19%. Ankle and foot fractures each contributed ~5% of total injuries.
Bentham et al. [13]	Cambridge, UK	2015-2022	Retrospective Radiology Review	340 pts (568 exams)	This radiological review noted that 80% of patients had extremity injuries and observed a significant rise in injuries after the October 2020 introduction of shared e-scooters.
Rauer et al. [15]	Bern, Switzerland	2017-2021	Retrospective Cohort	19 e-bike patients	Uniquely focusing on e-bikes, this study found that e-bike accidents produced a different, higher-velocity injury profile compared to typical e-scooter incidents.
Senel et al. [17]	Istanbul, Turkey	2019-2023	Retrospective Cohort	534 patients (247 injuries)	This cohort study reported fractures in 42.4% of patients, with the distal radius (wrist) being the most common fracture site at 17%, and also noted an increase in injuries during 2022-2023.
Shichman et al. [19]	Tel Aviv, Israel	2018-2020	Retrospective Cohort	3331 patients (2637 injuries)	This large-scale study identified a clear trend, showing a six-fold increase in e-scooter-related injuries presenting to the emergency department after shared services were introduced.
Ishmael et al. [20]	California, USA	2017-2019	Multicenter Retrospective Trauma Study	73 patients (42 lower limbs)	This study found a balanced distribution of trauma, with 43.8% of patients sustaining upper-extremity injuries and an equal 43.8% sustaining lower-extremity injuries, and noted a high surgical rate.
Vasara et al. [21]	Helsinki, Finland	2019-2021	Retrospective Cohort	434 patients (446 injuries)	This cohort study found that most injuries were of the upper limb. But due to a high-energy mechanism, all ankle injuries require surgical intervention.

Discussion

This systematic review provides compelling evidence that ankle fracture patterns resulting from e-scooter and e-bike use have become more common, severe and complex since the widespread adoption of these devices. Whilst attempting to focus on UK data, though limited, it shows a high proportion of lower-limb injuries in e-scooter trauma, mirroring global reports [3,4,6,7].

The evolution towards more severe fracture patterns can be attributed to several factors. First, the increased power and higher top speeds of newer generation e-scooters and e-bikes translate to greater kinetic energy during a crash [18]. Second, the typical mechanism of injury is a forward fall with the foot trapped in a position of rotation or forced dorsiflexion, which directly loads the ankle mortise with high, deforming forces [6]. The prevalence of Weber C, tri-malleolar, bimalleolar, pilon, and Maisonneuve patterns supports this high-energy rotational mechanism. Finally, a lack of rider experience, infrequent use of protective equipment and interactions with motor vehicles exacerbate the severity of these incidents and can result in open fractures. High travel speeds (>15 mph) were independently linked to more complex fractures [11]. The series by Ishmael et al. found e-scooter trauma bore many hallmarks of "high-energy trauma", a shift from pre-scooter years [20].

Significantly, the incidence of these injuries has climbed with device adoption. UK national data shows e-scooter collisions surged from 2020 onwards, consistent with the timing of rental scheme rollouts [6,7]. Similar trends are implied for e-bikes: trauma centres in London report dozens of severe e-bike cases in recent months [10,11,13]. Multiple sources highlight that the popularity of these devices has outpaced safety measures, leading to a growing burden of orthopaedic trauma [20,21]. AAOS, in their press release, noted a 23% annual rise in micromobility injuries, with nearly half of e-bike injuries occurring in 2022.

Our findings underscore the need for targeted prevention: speed restrictions, helmet/use of protective gear and public education may mitigate ankle fracture risk. Clinicians should be alert for complex fracture patterns in these patients. Trivedi et al. suggested using helmets and protective equipment whilst riding micromobility devices [23]. Currently, anyone aged 14 or over can ride an electric bike, with no requirement to have a licence or wear a helmet. E-bikes must have a maximum power output of 250 watts and cut out when they reach a speed of 15.5 mph.

Limitations

This review has limitations that have to be acknowledged. Several studies combine ankle injuries within the broader term of lower limb/lower extremity injuries, thus reducing the granularity of fracture type analysis. The heterogeneity amongst studies was notable. There is a potential publication bias towards more severe injuries. Furthermore, the conflation of e-scooter and e-bike data, while necessary due to the current literature, may obscure nuanced differences in their injury mechanisms. Temporal and geographic variation in e-bike and e-scooter adoption could affect generalizability. Some countries adopted micromobility earlier, leading to differences in user behaviour, infrastructure safety and injury trends. Anecdotally, many included studies began data collection after scooters were first introduced, so a true "before vs. after" comparison is lacking. Nonetheless, the evidence clearly indicates an increasing incidence of orthopaedic injuries in the micromobility era.

## Conclusions

This systematic review shows that e-scooter and e-bike accidents, while causing predominantly upper-extremity injuries, do involve substantial ankle fractures. E-scooter crashes often result in pronation-external rotation ankle fractures, frequently with posterior or medial malleolar fragments, and open wounds. E-bike crashes less commonly injure the ankle, but may cause severe bimalleolar fracture dislocations. Clinicians should maintain a high index of suspicion for complex ankle fractures in micromobility injuries. Given the upward trend, there is a pressing need for preventive strategies (e.g. speed limits, protective gear mandates) and public education to mitigate the growing orthopaedic burden. As usage continues to climb, public health measures (helmet and footwear advice, speed limits) and clinical vigilance are needed to manage the rising public health concern of ankle trauma.
